# Examining breast cancer screening recommendations in Canada: The projected resource impact of screening among women aged 40–49

**DOI:** 10.1177/09691413241267845

**Published:** 2024-08-06

**Authors:** Robert B Basmadjian, Yibing Ruan, John M Hutchinson, Matthew T Warkentin, Oguzhan Alagoz, Andrew Coldman, Darren R Brenner

**Affiliations:** 1 Department of Oncology, Cumming School of Medicine, 2129 University of Calgary, Calgary, Alberta, Canada; 2 Department of Industrial and Systems Engineering, University of Wisconsin-Madison, Madison, WI, USA; 3 206022 Carbone Cancer Center, University of Wisconsin-Madison, Madison, WI, USA; 4 8162 British Columbia Cancer Control Research, Vancouver, British Columbia, Canada; 5 Department of Community Health Sciences, Cumming School of Medicine, 2129 University of Calgary, Calgary, Alberta, Canada

**Keywords:** Breast cancer, early detection, epidemiology, healthcare resources, screening

## Abstract

**Objective:**

To quantify the resource use of revising breast cancer screening guidelines to include average-risk women aged 40–49 years across Canada from 2024 to 2043 using a validated microsimulation model.

**Setting:**

OncoSim-Breast microsimulation platform was used to simulate the entire Canadian population in 2015–2051.

**Methods:**

We compared resource use between current screening guidelines (biennial screening ages 50–74) and alternate screening scenarios, which included annual and biennial screening for ages 40–49 and ages 45–49, followed by biennial screening ages 50–74. We estimated absolute and relative differences in number of screens, abnormal screening recalls without cancer, total and negative biopsies, screen-detected cancers, stage of diagnosis, and breast cancer deaths averted.

**Results:**

Compared with current guidelines in Canada, the most intensive screening scenario (annual screening ages 40–49) would result in 13.3% increases in the number of screens and abnormal screening recalls without cancer whereas the least intensive scenario (biennial screening ages 45–49) would result in a 3.4% increase in number of screens and 3.8% increase in number of abnormal screening recalls without cancer. More intensive screening would be associated with fewer stage II, III, and IV diagnoses, and more breast cancer deaths averted.

**Conclusions:**

Revising breast cancer screening in Canada to include average-risk women aged 40–49 would detect cancers earlier leading to fewer breast cancer deaths. To realize this potential clinical benefit, a considerable increase in screening resources would be required in terms of number of screens and screen follow-ups. Further economic analyses are required to fully understand cost and budget implications.

## Introduction

Breast cancer is the most diagnosed malignancy and second-most common cause of cancer death among Canadian women.^1^ The introduction of organized breast cancer screening programs, coupled with advancements in medical technologies and treatments, have reduced breast cancer age-standardized mortality rates by 47%—from 42.7 per 100,000 women in 1986 to 22.5 per 100,000 women in 2020.^1,2^ In their 2018 recommendations, the Canadian Task Force for Preventive Health suggest that women aged 50 to 74 years at average risk of developing breast cancer receive screening every 2 to 3 years.^3^ Implementation of cancer screening programs is overseen by provinces and territories, most of which recommend screening asymptomatic individuals at average risk with a mammogram every 2 years starting at age 50 until age 74.^4^

In 2023, the Cancer Intervention and Surveillance Modeling Network (CISNET) Breast Cancer Working Group used microsimulation modeling to examine the impact of alternate screening programs for women in their 40s on annual and lifetime cancer outcomes in the US.^5^ Their report suggested reduced breast cancer mortality and improved life expectancy among average-risk women with biennial screening starting at age 40 or 45. Subsequently, the US Preventive Services Task Force (USPSTF) issued final recommendations suggesting that biennial breast cancer screening for women at average risk for breast cancer be considered between ages 40 and 74, lowering the previously recommended starting age of 50.^6^ A similar microsimulation model, OncoSim, exists in Canada and has been validated to represent the breast cancer incidence and mortality, as well as the screening efficacy, in Canada.^7^ Recently, Yaffe and Mainprize used this model to demonstrate mortality reductions and additional life-years gained by starting annual or biennial screening at age 40 rather than age 50 in Canada.^8^

While the clinical benefit of screening women aged 40 to 49 years has been established, revising screening guidelines will inevitably increase demands on provincial and territorial healthcare systems, which have yet to be investigated. Some jurisdictions in Canada already accept average-risk women under the age of 50 for routine breast cancer screening. For example, both Prince Edward Island (PEI) and Nova Scotia offer annual screening for average-risk women aged 40 to 49, whereas Alberta and British Columbia currently offer biennial screening starting at age 45 and age 40, respectively. However, other jurisdictions may be prone to resource restrictions and find it challenging to revise guidelines. Quantifying the projected burden of increased screening demands may inform policy makers about what resources are required to support organized screening among average-risk women in their 40s in jurisdictions where it is currently not offered. To address this need, we used the OncoSim microsimulation model tool to estimate 20-year resource impacts of alternate breast cancer screening programs among women aged 40 to 49 in the Canadian population. The alternate programs included annual or biennial screening for average-risk women aged 40 to 49 or 45 to 49, followed by biennial screening for ages 50 to 74, to examine the resource impact of existing screening programs like those in Nova Scotia, PEI, Alberta, and British Columbia.

## Methods

### OncoSim-Breast microsimulation model

The detection and progression of breast cancer outcomes were simulated using the breast cancer modules in OncoSim-Breast (v3.6.2.5), a cancer microsimulation modeling tool developed by the Canadian Partnership Against Cancer in collaboration with Statistics Canada. OncoSim simulates individuals to mimic the demographic characteristics of the Canadian population. The development, application, and validation of the OncoSim-Breast models have been described by Yong et al.^7^

OncoSim-Breast simulates the onset, growth, and spread of invasive tumors and ductal carcinoma *in situ* (DCIS) (Figure S1). The simulation of natural history was calibrated to match Canadian cancer incidence and mortality data. OncoSim-Breast was inspired by the University of Wisconsin breast cancer microsimulation model, one of the six CISNET breast cancer models.^9,10^ OncoSim-Breast captures the benefits of screening on breast cancer survival using the lead time that was calibrated from observed survival data. Staging is based on the American Joint Committee on Cancer's classification of tumor size (T), nodal status, (N), and metastasis (M). To account for geographic variation, the survival outcomes were adjusted for each province and territory using relative risks and calibrated to match province-specific cancer mortality data in the Canadian Cancer Registry. The effects of projected population growth and aging demographics are modeled, contributing to an annual increase in the number of breast cancers. The model follows women to death (maximum allowed age is 119), and results were reported from calendar years 2015 to 2051.

### Screening scenarios

We simulated five screening scenarios as described in Table 1. The Status quo (SQ) scenario emulated current breast cancer screening guidelines in Canada (biennial screening for ages 50–74) and was used as the baseline/referent group. We compared SQ against four screening scenarios of interest: A40, revise current guidelines to include annual screening among average-risk women aged 40–49; B40, revise current guidelines to include biennial screening among average-risk women aged 40–49; A45, revise current guidelines to include annual screening among average-risk women aged 45–49; and B45, revise current guidelines to include biennial screening among average-risk women aged 45–49.

**Table 1.** Description of screening scenarios in the 40–49 and 45–49 age groups starting in 2024.

Scenario	Description	Comments
SQ	Status quo	Organized screening targets average-risk women aged 50–74 years only; ∼25% of women aged 40–49 years receive annual screening
A40	Annual 40–49	Starting in 2024, organized screening enrolls average-risk women aged 40–49 years for screening every year; participation rate: 50%, 70%, 100%
B40	Biennial 40–49	Starting in 2024, organized screening enrolls average-risk women aged 40–49 years for screening every 2 years; participation rate: 50%, 70%, 100%
A45	Annual 45–49	Starting in 2024, organized screening enrolls average-risk women aged 45–49 years for screening every year; participation rate: 50%, 70%, 100%
B45	Biennial 45–49	Starting in 2024, organized screening enrolls average-risk women aged 45–49 years for screening every 2 years; participation rate: 50%, 70%, 100%

For all scenarios, current guidelines were followed until the end of 2023. Then, on January 1st, 2024, screening guidelines were revised to include 40–49 and 45–49 age groups for routine annual and biennial screening, depending on the scenario. Participation rates in screening scenarios were determined by a weighted average of high-risk women and average-risk women. Oncosim-Breast assumes 25% of women are high risk for breast cancer and includes those with an inherited genetic predisposition (*BRCA1/2*) or a first-degree or second-degree family history of breast cancer.^7^ The remaining 75% are assumed to be average risk.^7^ In all scenarios, including the SQ, we assumed 25% uptake of annual screening among women aged 40–49 due to high-risk status and opportunistic screening among average-risk women from 2015 to 2051. We also assumed 100% of women aged 50–74 enrolled in biennial screening. The re-screen rate was assumed to be 100% for high-risk women and 63% for average-risk women. Output was generated for all scenarios when the target participation rate in the new screening programs among women aged 40–49 years was 50%, 70%, and 100%. We focused on results when participation was 50% to understand the real-world implications of our screening scenarios.

### Outcomes modeled

The primary resource outcomes modeled for the A40, B40, A45, and B45 scenarios relative to SQ included number of additional screens, abnormal screening recalls where cancer was not diagnosed, number of biopsies, and number of negative biopsies. The number of total and negative biopsies refer to those resulting from a positive screen. We also modeled clinical outcomes, including the number of additional invasive breast cancers, breast cancers detected by screening (invasive and DCIS), and averted breast cancer deaths. The number of invasive breast cancers refers to all diagnoses in Canada regardless of whether screening was performed. The cumulative number of additional outcomes between 2024 and 2043 are reported in tables and visualized using bar plots. Relative percent change of outcomes between the SQ and alternate scenarios was also calculated by subtracting the outcome value in the alternate scenario from that of the SQ scenario, dividing by the value in the SQ scenario, and multiplying by 100%. We also report the rate of these outcomes per 100,000 screens for all scenarios. To quantify the demand for resources upon program implementation, we calculated the number of additional outcomes and relative percent change from 2023 to 2024 for all scenarios. The cumulative number of DCIS and invasive breast cancer cases between 2024 and 2043 by stage (0, I, II, III, IV) were estimated. Finally, we estimated the efficiency of additional screening by plotting the number of averted breast cancer deaths against number of additional screens.

## Results

### Total number of additional outcome events relative to SQ

#### Screening resource outcomes

**Figure 1.**
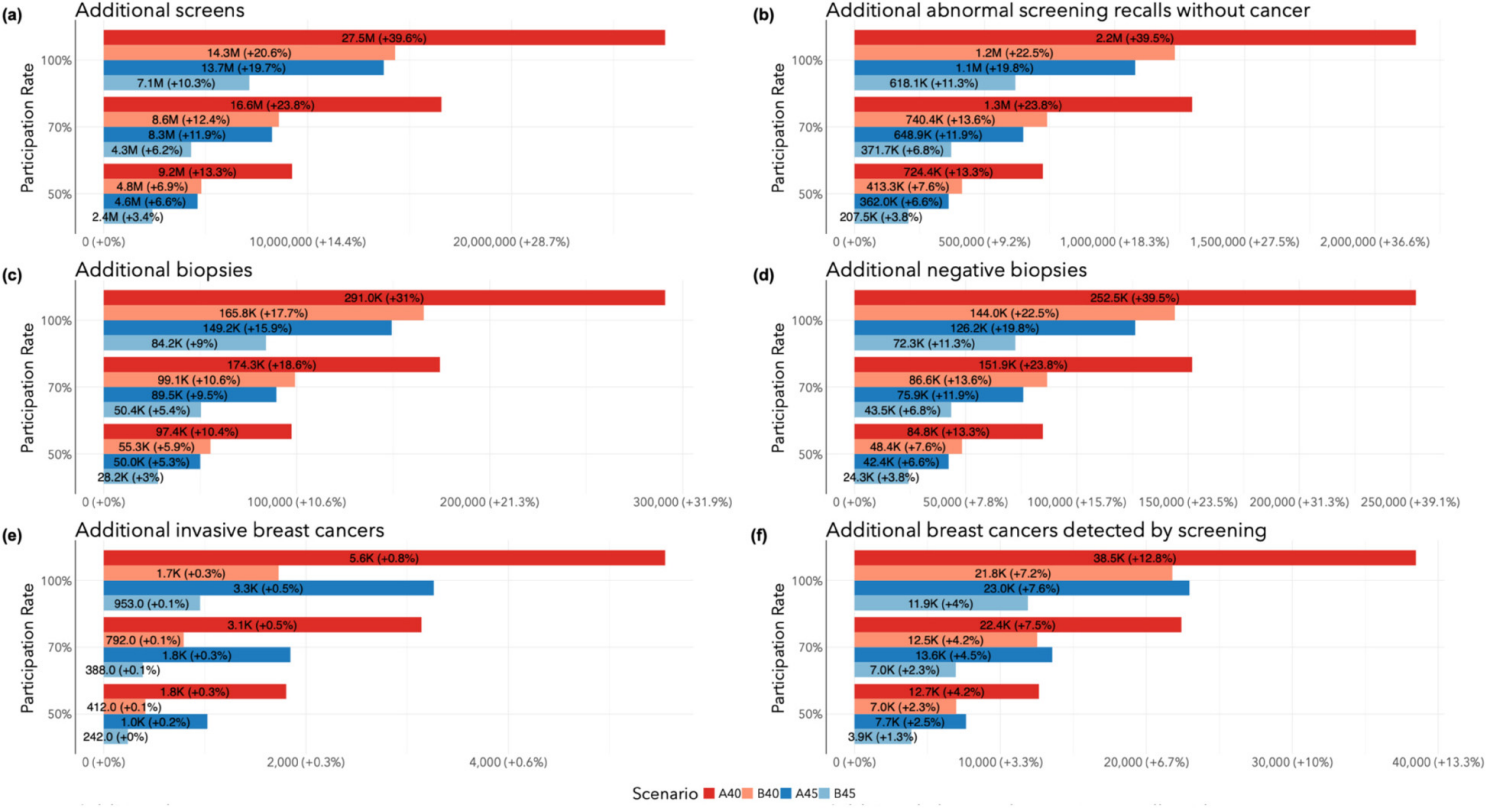
Total number of additional outcome events (2024–2043) by target participation rate in screening program change relative to Status quo: (a) number of screens; (b) abnormal screening recalls without cancer; (c) biopsies; (d) negative biopsies; (e) invasive breast cancers; (f) breast cancers detected by screen.

The total number of additional screens, abnormal screening recalls without cancer, biopsies, and negative biopsies relative to the SQ scenario in Canada from 2024 to 2043 are illustrated in Figure 1(a)–(d) and calculated from values in Table 2 and Table S1. The A40 program would result in an additional 9.24 M screens (+13.3%), 724 K abnormal screening recalls without cancer (+13.3%), 97.4 K biopsies (+10.4%), and 84.8 K negative biopsies (+13.3%). The B40 program would result in an additional 4.8 M screens (+6.9%), 413 K abnormal screening recalls without cancer (+7.6%), 55.3 K biopsies (+5.9%), and 48.4 K negative biopsies (+7.6%). The A45 program would result in an additional 4.61 M screens (+6.6%), 362 K abnormal screening recalls without cancer (+6.6%), 50 K biopsies (+5.3%), and 42.4 K negative biopsies (+6.6%). Finally, the B45 program would result in an additional 2.4 M screens (+3.4%), 207 K abnormal screening recalls without cancer (+3.8%), 28.2 K biopsies (+3.0%), and 24.3 K negative biopsies (+3.8%). The number of additional screens, abnormal screening recalls without cancer, biopsies, and negative biopsies when target participation rates were set to 70% and 100% are included in Table S2.

**Table 2.** Total number of outcome events among all women in Canada from 2024 to 2043 when the target participation rate of the A40, B40, A45, and B45 scenarios is 50%.

Scenario	Women screened	Screens	Abnormal screening recalls without cancer	Biopsies	Negative biopsies	Invasive breast cancers	Breast cancers detected by screening	Cancer deaths
SQ	5,910,283	69,573,861	5,461,199	939,498	638,960	660,943	300,538	125,836
A40	6,684,096	78,816,485	6,185,564	1,036,900	723,711	662,747	313,189	124,934
B40	6,684,096	74,373,190	5,874,533	994,839	687,320	661,355	307,519	125,254
A45	6,305,809	74,180,113	5,823,189	989,508	681,313	661,969	308,195	125,297
B45	6,305,809	71,972,882	5,668,683	967,694	663,236	661,186	304,458	125,399

#### Clinical outcomes

The total numbers of additional invasive breast cancers and breast cancers detected by screening relative to SQ in Canada from 2024 to 2043 are calculated from values in Table 2 and Table S1, and illustrated in Figure 1(e) and (f). The A40 program would result in 1804 more invasive breast cancers (+0.27%) and 12.7 K more screen-detected cancers (+4.2%). The B40 program would result in 412 more invasive cancers (+0.06%) and 6981 more screen-detected cancers (+2.3%). The A45 program would result in 1026 more invasive cancers (+0.16%) and 7657 more screen-detected cancers (+2.5%). Finally, the B45 program would result in 242 more invasive cancers (+0.04%) and 3920 more screen-detected cancers (+1.3%). The number of additional invasive cancers in the revised programs relative to SQ was driven by screen-detected invasive cancers among women aged 40 or 45–49 near the end of the observation period that would not have been diagnosed otherwise.

All screening programs resulted in fewer breast cancer deaths in Canada from 2024 to 2043 relative to SQ (Table 2). The A40, B40, A45, and B45 screening programs would result in 902 (−0.72%), 582 (−0.46%), 538 (−0.43%), and 436 (−0.35%) averted breast cancer deaths, respectively. The numbers of additional invasive breast cancers and breast cancers detected by screening, and breast cancer deaths averted when target participation rates were set to 70% and 100% are included in Table S2.

**Figure 2.**
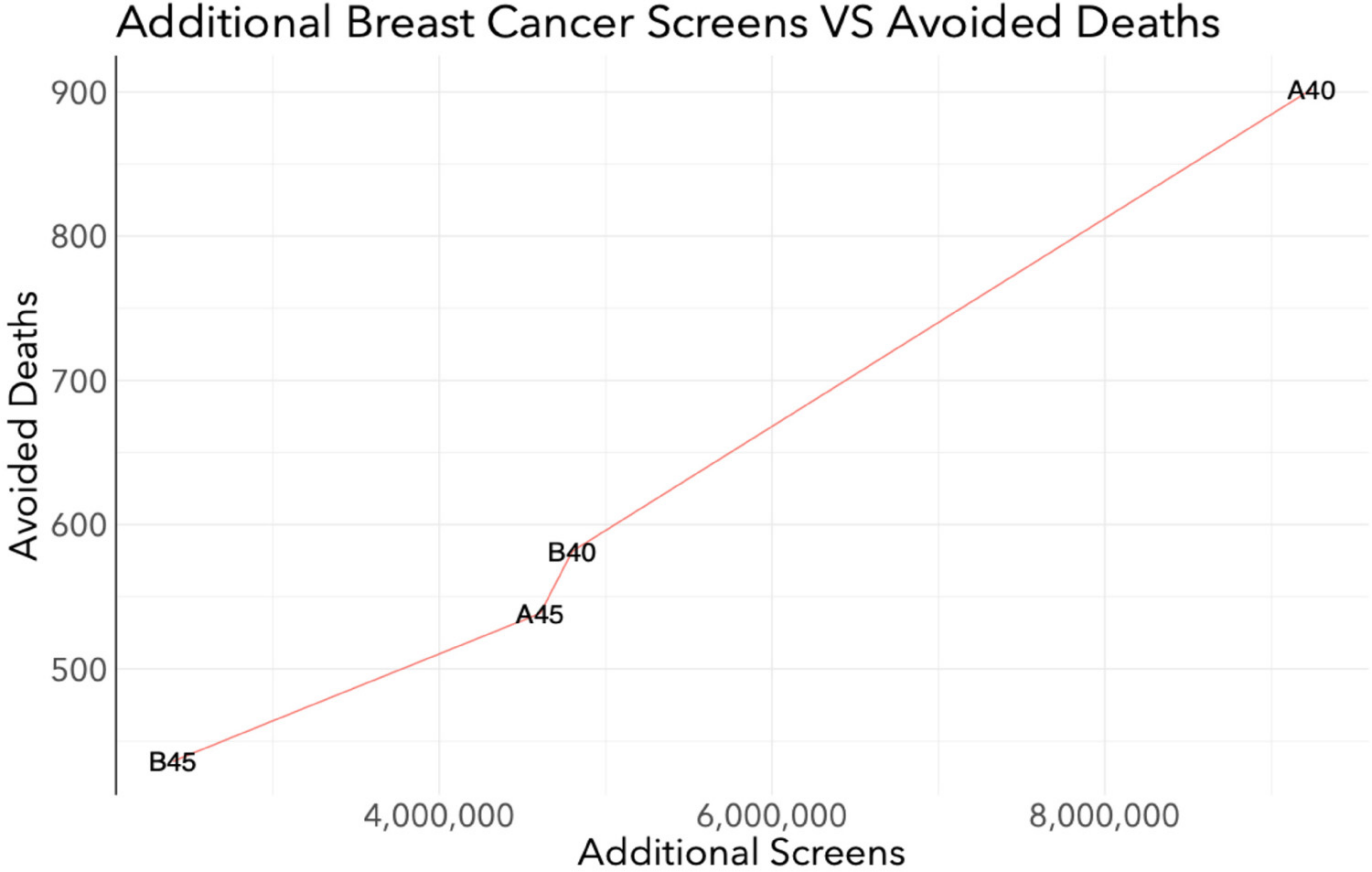
Efficiency of averting breast cancer deaths: breast cancer deaths averted vs. number of screens among all women in Canada from 2024 to 2043 when the target participation rate of the A40, B40, A45, and B45 scenarios is 50%.

When averted cancer deaths are plotted against the additional number of screens, there was a notable inflection in the line between the A45 and B40 scenarios (Figure 2). This indicated that a minimal increase in additional number of screens would result in a large increase in averted deaths in the B40 program relative to the A45 program.

### Outcome rates per 100,000 screens

The total number of outcome events per 100,000 screens in Canada from 2024 to 2043 for all scenarios is calculated from values in Table 2 and presented in Table 3. In general, the rates of breast cancers detected by screening, invasive breast cancers, biopsies, and cancer deaths were highest in the SQ. The A40 scenario had the lowest rates for all outcomes. The rates of abnormal screening recalls without cancer and negative biopsies were highest in the B40 scenario. The rate of breast cancer death was 158.5 per 100,000 screens in the A40 scenario, 168.4 per 100,000 screens in the B40 scenario, 168.9 per 100,000 screens in the A45 scenario, and 174.2 per 100,000 screens in the B45 scenario.

**Table 3.** Total number of outcome events per 100,000 screens among all women in Canada from 2024 to 2043 when the target participation rate of the A40, B40, A45, and B45 scenarios is 50%.

Scenario	Abnormal screening recalls without cancer	Biopsies	Negative biopsies	Invasive breast cancers	Breast cancers detected by screening	Cancer deaths
SQ	7849	1350	918.4	950	432	180.9
A40	7848	1316	918.2	841	397	158.5
B40	7899	1338	924.2	889	413	168.4
A45	7850	1334	918.5	892	416	168.9
B45	7876	1345	921.5	919	423	174.2

### Resource demands at program implementation

Compared with 2023, the implementation of revised screening guidelines in 2024 would result in a surge of resource use (Table 4). The A40 and B40 would result in an additional 742 K (+25.1%) screens, 117 K (+51.1%) abnormal screening recalls without cancer, 16.1 K (+40.6%) biopsies, 13.7 K (+51.1%) negative biopsies, and 2356 (+18.4%) detected breast cancers. The A45 and B45 would result in an additional 407 K (+13.8%) screens, 60.5 K (+26.3%) abnormal screening recalls without cancer, 8663 biopsies (+21.8%), 7077 (+26.3%) negative biopsies, and 1586 (+12.4%) detected breast cancers. Finally, the SQ scenario would result in an additional 89.9 K (+3.0%) screens, 6606 (+2.9%) abnormal screening recalls without cancer, 1109 biopsies (+2.8%), 773 (+2.9%) negative biopsies, and 1586 (+12.4%) detected breast cancers. Annual outcome plots for all scenarios and participation rates are presented in Figures S2-S4.

**Table 4.** Absolute and relative percent change in outcome events from 2023 to 2024 among all women in Canada when the target participation rate of the A40, B40, A45, and B45 scenarios is 50%.

Scenario	Screens	Abnormal screening recalls without cancer	Biopsies	Negative biopsies	Breast cancers detected by screening
SQ^a^	89,883 (+3.0%)	6606 (+2.9%)	1109 (+2.8%)	773 (+2.9%)	337 (+2.6%)
A40	742,176 (+25.1%)	117,496 (+51.1%)	16,103 (+40.6%)	13,747 (+51.1%)	2356 (+18.4%)
B40	742,176 (+25.1%)	117,496 (+51.1%)	16,103 (+40.6%)	13,747 (+51.1%)	2356 (+18.4%)
A45	406,828 (+13.8%)	60,486 (+26.3%)	8663 (+21.8%)	7077 (+26.3%)	1586 (+12.4%)
B45	406,828 (+13.8%)	60,486 (+26.3%)	8663 (+21.8%)	7077 (+26.3%)	1586 (+12.4%)

^a^ Despite there being no changes to screening in the SQ scenario, the number of women and resources increases as a function of population growth.

### Total number of ductal carcinoma *in situ* and invasive breast cancer cases by stage

**Figure 3.**
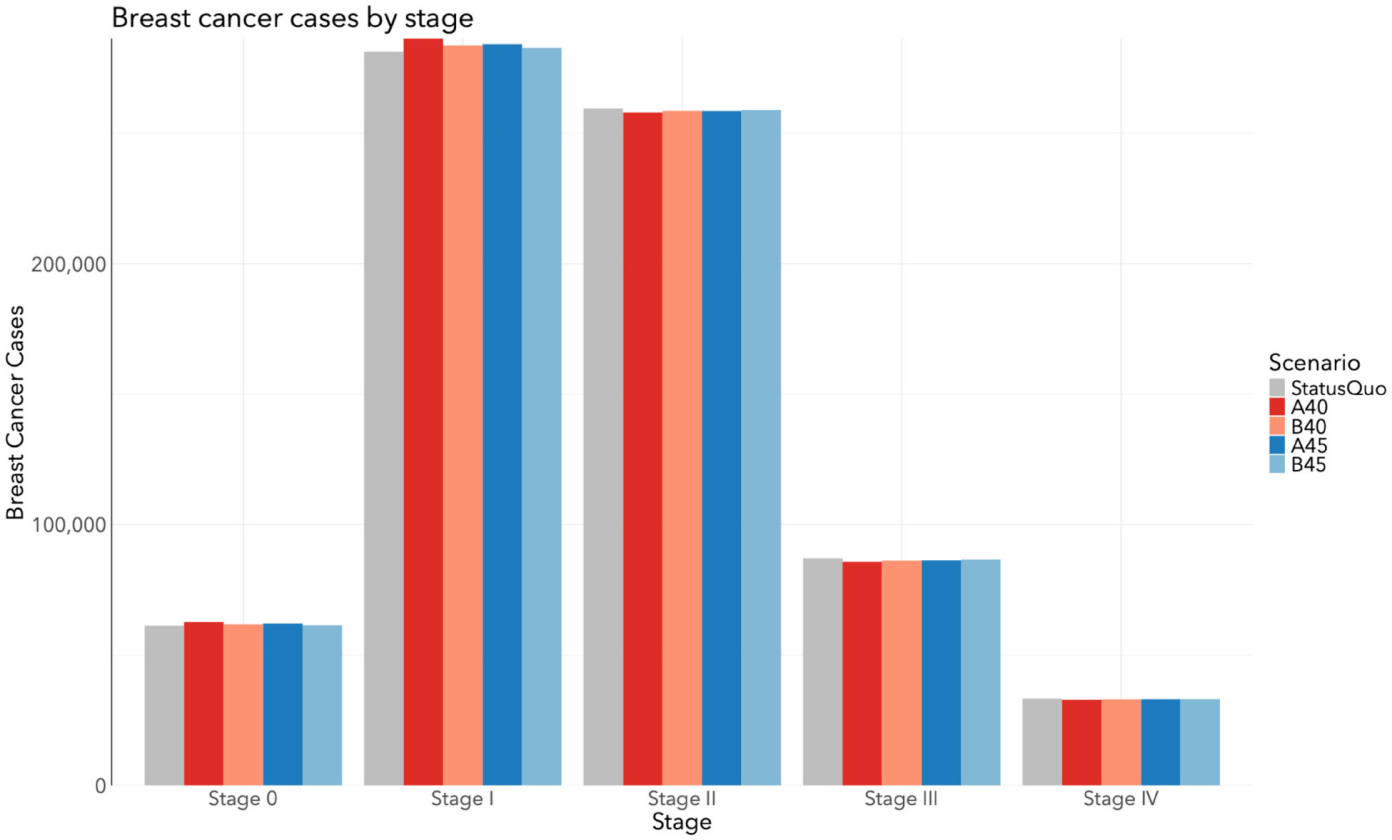
Total number of invasive breast cancer and DCIS cases by stage at diagnosis among all women in Canada from 2024 to 2043 when the target participation rate of the A40, B40, A45, and B45 scenarios is 50%.

**Figure 4.**
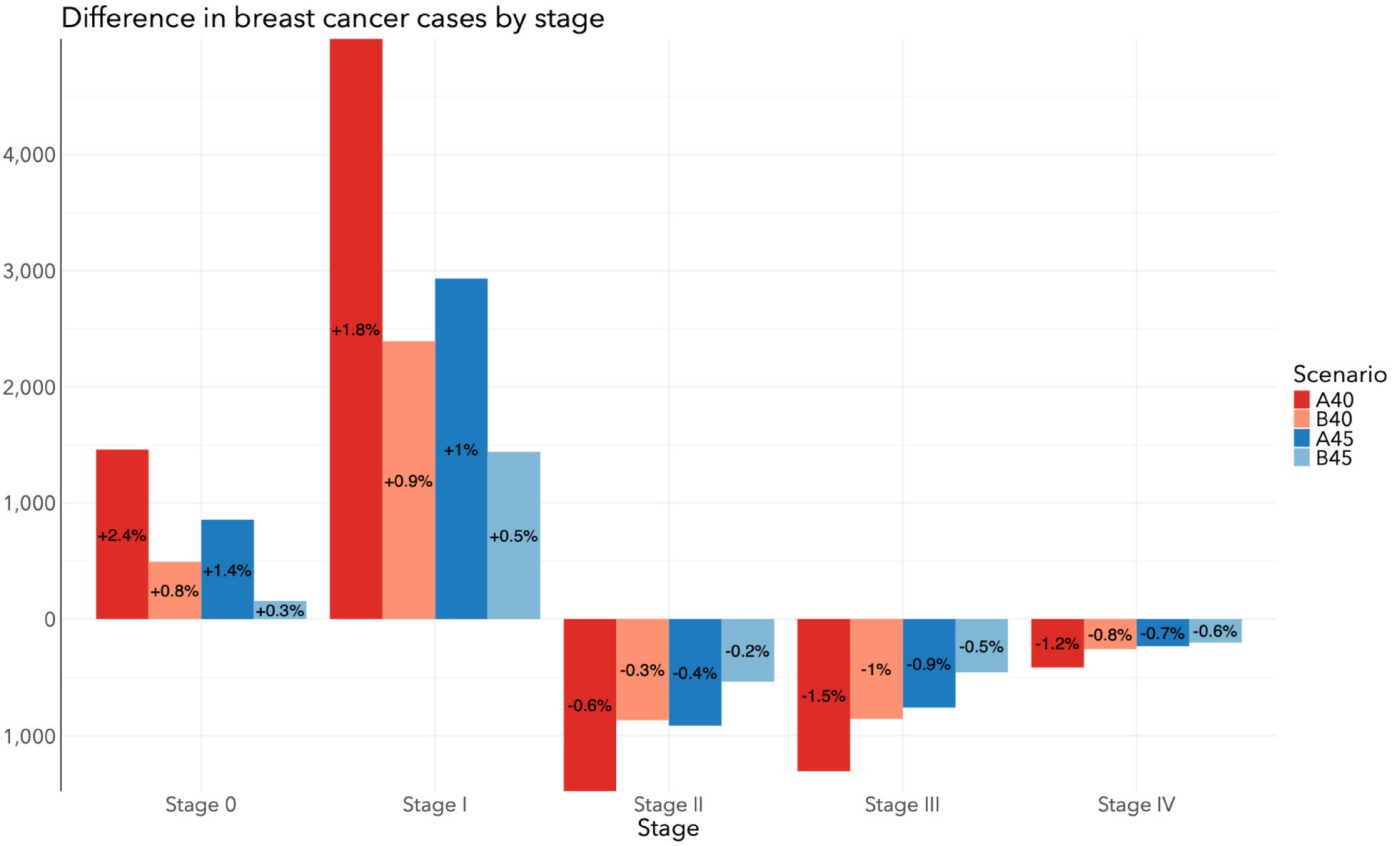
Difference in stage-specific breast cancer cases between screening program change and Status quo among all women in Canada from 2024 to 2043 when the target participation rate of the A40, B40, A45, and B45 scenarios is 50%.

The total number of DCIS and invasive breast cancer cases by stage for each screening scenario in Canada from 2024 to 2043 is depicted in Figure 3. Relative to SQ, all scenarios resulted in additional DCIS and stage I diagnoses, and fewer stage II, III, and IV diagnoses (Figure 4). The A40 scenario resulted in 1475 fewer stage II (−0.57%), 1303 fewer stage III (−1.5%), and 415 fewer stage IV diagnoses (−1.2%). The B40 scenario resulted in 867 fewer stage II (−0.33%), 856 fewer stage III (−0.98%), and 258 fewer stage IV diagnoses (−0.77%). The A45 scenario resulted in 913 fewer stage II (−0.35%), 759 fewer stage III (−0.87%), and 234 fewer stage IV diagnoses (−0.71%). The B45 scenario resulted in 538 fewer stage II (−0.21%), 458 fewer stage III (−0.53%), and 202 fewer stage IV diagnoses (−0.61%). Table 5 includes frequencies and proportions of stage-specific cancers by screening scenario. In general, proportions were consistent across all scenarios.

**Table 5.** Frequency and proportions of invasive breast cancer and DCIS by stage at diagnosis among all women in Canada from 2024 to 2043 when the target participation rate of the A40, B40, A45, and B45 scenarios is 50%.

Stage	SQ, *N*(%)	A40, *N*(%)	B40, *N*(%)	A45, *N*(%)	B45, *N*(%)
Stage 0	61,214 (8.48)	62,673 (8.64)	61,706 (8.53)	62,070 (8.57)	61,370 (8.49)
Stage I	281,197 (38.94)	286,194 (39.45)	283,590 (39.22)	284,129 (39.24)	282,637 (39.12)
Stage II	259,398 (35.92)	257,922 (35.55)	258,531 (35.76)	258,485 (35.7)	258,859 (35.83)
Stage III	87,051 (12.05)	85,748 (11.82)	86,195 (11.92)	86,292 (11.92)	86,593 (11.98)
Stage IV	33,298 (4.61)	32,883 (4.53)	33,039 (4.57)	33,063 (4.57)	33,096 (4.58)

## Discussion

This microsimulation modeling study quantified the resource needs to support revising breast cancer screening guidelines to include women aged 40–49 in Canada. As expected, more intensive screening program revisions (increasing age range and screening frequency) would be accompanied by larger numbers of screens, abnormal screening recalls without cancer, biopsies, negative biopsies, screen-detected cancers, and averted deaths compared with current screening guidelines. The most intensive revision (A40 scenario) would result in a 13% increase in number of screens, abnormal screening recalls without cancer, and negative biopsies whereas the least intensive revision (B45 scenario) would result in an approximate 4% increase in these metrics. Despite requiring the most resources, the A40 scenario would avert the most breast cancer deaths, more than double that would be averted with the B45 scenario. The B40 scenario, which emulated the USPSTF's new screening recommendations, would result in a slightly greater use of resources and fewer screen-detected cancers and cancer deaths than the A45 scenario.

From the perspective of the publicly funded Canadian healthcare system, it is essential to consider the substantial resource use of revised screening guidelines with other aspects of breast cancer management, including diagnosis and treatment. Our findings show an immediate burden of screening on healthcare upon revising guidelines in 2024. The major implication of this is that many capacity restraints and costs would be allocated to the beginning of the program whereas benefits, like averted deaths and life-years gained, would not be observed until later. For example, the surge of abnormal screening recalls without cancer is expected since many women in their 40s would receive their first screen, which is more likely to be associated with an abnormal screening recall without cancer compared with subsequent screens because of the lack of prior screens for comparison.^11,12^ Further, women in their 40s are more likely to have dense breast tissue, which makes it difficult for radiologists to spot cancers on mammogram readings.^13^ However, OncoSim-Breast does not currently account for breast density so our projections may underestimate abnormal screening recalls without cancer. This would lead to the corresponding surge in total and negative biopsies to establish a “baseline” breast examination. After this point, however, there would be a sharp decline in the following year, then a steady rise again as more women in their 40s enter the screening program with population growth over time. There would also be an immediate surge of screen-detected cancers, reflecting prevalent cancers among women in their 40s, which would not have been previously detected without screening.

Revising screening guidelines would result in fewer breast cancers detected per 100,000 screens compared to current guidelines, which was expected because breast cancer risk increases with age and is more common among women aged 50–74 than 40–74. Detecting higher absolute numbers would drive resource demands and costs of treating more breast cancers but could be offset by detecting cancers at an earlier stage. Our findings show the A40 scenario would result in the largest stage shift (fewest stage III/IV and most DCIS/stage I) and helps explains why this scenario would avert the most deaths. Later stage cancers involve more complex, intensive, and expensive therapies with long durations and lead to morbidities that would require further clinical care. Wilkinson et al.^14^ performed a comprehensive analysis to estimate the per-case cost of breast cancer treatments by stage and molecular subtype in Ontario and considered diagnostic procedures, pathology, surgery, radiation, systemic therapy, inpatient, emergency, home care and palliative care costs. Stage IV costs were as high as $516,415 (CAD) per case, 10.9 times the cost of stage I cancers and 35.6 times the cost of DCIS.^14^ Unfortunately, the authors did not model costs for treatment morbidities or treatment for recurrent disease. Other population-based studies examining resource use and costs of treating breast cancer in Ontario found patients with stage IV disease utilized significantly more emergency, home care, and inpatient services, reflecting intensified care requirements.^15–17^ This suggests the A40 scenario would alleviate the most resource and cost burdens of treating breast cancer on the healthcare system. However, this needs to be confirmed by an economic analysis that accounts for the treatment of recurrent disease and morbidities to determine the cost-effectiveness of treating an additional 1459 DCIS cases and 4997 stage I cancers.

Interestingly, the A45 scenario would detect a higher number of breast cancers than the B40 scenario but it would avert fewer stage III and IV cancers. There are two implications from these findings. The first is that screening women at an earlier age likely detects fast-growing cancers that would otherwise be later stage and symptomatic. The second is that fewer resources and costs would be allocated to treating breast cancers in the B40 scenario compared with the A45 scenario while having greater benefit (more averted deaths).

Although revising screening guidelines to include women aged 40–49 would alleviate the healthcare system of treating fewer later stage cancers, a greater proportion of resources required would be attributed to screening itself compared with current guidelines. This is supported by the fact that increasing the number of screens performed resulted in more screening recalls, most of which would be negative for the presence of cancer. Surprisingly, the A40 scenario resulted in the lowest rates of abnormal screening recalls without cancer and negative biopsies per 100,000 screens. Given that the A40 scenario also had the lowest detection rate, it would be the most efficient at ruling out disease, even compared with current guidelines. In other words, each screen performed in the A40 scenario would result in the lowest likelihood of detecting cancer, recalling women for addition screening, undergoing biopsy procedures, and cancer death. The B40 scenario had the highest rates of abnormal screening recalls without cancer and negative biopsies. Therefore, when compared with the A45 scenario, averting slightly more cancer deaths in the B40 scenario would come at the cost of recalling more women and performing more biopsies, but less resources would be required for treating cancers, as stated previously. When comparing averted cancer deaths against additional number of screens, the slope of the line is highest between the A45 and B40 scenarios, suggesting that a similar additional number of screens on younger women averts more deaths.

Mittmann et al.^18^ used microsimulation modeling to evaluate the cost-effectiveness of breast cancer screening programs in Canada and demonstrated that more intensive screening increased the proportion for which screening contributed to total costs. For biennial screening ages 50–74 (like our SQ/current guidelines), screening contributed 44% to the total costs, whereas biennial screening ages 40–74 (like our B40 scenario) and annual screening ages 40–49 followed by biennial screening ages 50–74 (like our A40 scenario) contributed to 53% and 59% of total costs, respectively. This agrees with our findings of more resources being allocated to screening itself.

Yaffe and Mainprize also conducted a simulation study using the OncoSim-Breast model to estimate the impact of breast screening starting at age 40 in Canada.^8^ They observed fewer breast cancer deaths per 1000 women in their biennial 40–74 and annual 40–49/biennial 50–74 scenarios, relative to the biennial 50–74 scenario. The mortality reductions of these scenarios were accompanied by higher recall and negative biopsy rates per 1000 women, which were congruent with our findings. However, there are key differences between our analyses that should be noted. First, Yaffe and Mainprize restricted their analysis to a cohort of 1.53 M Canadian women born in 1975 whereas we do not restrict on birth cohort. The cohort-based modeling approach is more appropriate for evaluating the clinical benefits of revised screening guidelines. Our study leveraged a population-based modeling approach to more appropriately estimate the resources used across the entire simulated Canadian population prevalent each year of the analyses. Second, they assume 100% participation of cohort members in the screening regimen – that all women receive their examinations at the indicated protocol, which is acknowledged as a limitation.^8^ We examined outcomes at different participation rates (50%, 70%, and 100%) among women aged 40–49 years, and assumed a re-screen rate of 63%. Therefore, our study estimates more likely reflect real-world screening conditions in the Canadian population.

A major strength of our analysis is that the OncoSim-Breast model is developed using Canadian data and validated to represent the underlying Statistic Canada population, mortality and Canadian Cancer Registry data. Another strength is that we varied participation rates in screening scenarios among women aged 40–49 years. This accounts for the fact that participation in the real world will be driven by personal values and beliefs on the benefits and harms of screening. Further, our referent group models current screening guidelines with imperfect re-screen rates, allowing us to examine more realistic impacts of revising screening to include women aged 40–49 than other studies. Our study also includes noteworthy limitations. The first is that national-level analyses in OncoSim-Breast pool outcomes across provinces and territories and do not easily report province- or territory-specific outcomes. A second limitation is that we did not assess the economic impact of our screening scenarios, which will be an important factor in decision making for revised screening guidelines.

## Conclusion

In conclusion, lowering the minimum screening age to 40 and 45 years among average-risk women would shift resource allocation from treating later stage breast cancers towards performing more screens and biopsies. Our work includes helpful information for decision makers at the provincial and territorial level regarding the feasibility of implementing organized breast cancer screening programs. In addition to quantifying cumulative resource use over 20 years, we project year-by-year resource use to demonstrate when the healthcare system would face the most burden and whether jurisdictions could accommodate the demand. While some jurisdictions in Canada already accept average-risk women under the age of 50 for routine breast cancer screening, others may be prone to resource restrictions or face challenges with participation and adherence among women aged 50–74. Among some provinces that have revised guidelines, screening is made available to women in the 40s through self-referral but they are not actively recruited by the provinces. Leaving the decision to screen between ages 40 and 49 to the individual may alleviate some resource capacity issues on the provincial healthcare systems resulting from revised guidelines. Given the noted mortality reductions and life-years gained, jurisdictions could consider accepting average-risk women in their 40s who choose to be screened where it is currently not offered.

## Supplemental Material

sj-docx-1-msc-10.1177_09691413241267845 - Supplemental material for Examining breast cancer screening recommendations in Canada: The projected resource impact of screening among women aged 40–49 Supplemental material, sj-docx-1-msc-10.1177_09691413241267845 for Examining breast cancer screening recommendations in Canada: The projected resource impact of screening among women aged 40–49 by Robert B Basmadjian, Yibing Ruan, John M Hutchinson, Matthew T Warkentin, Oguzhan Alagoz, Andrew Coldman and Darren R Brenner in Journal of Medical Screening
