# Supplementary material for: Examining breast cancer screening recommendations in Canada: The projected resource impact of screening among women aged 40–49
Source: J Med Screen. 2024 Aug 6;32(1):35–43. doi: 10.1177/09691413241267845 (PMC11869510; doi:10.1177/09691413241267845)
Supplement: sj-docx-1-msc-10.1177_09691413241267845 - Supplemental material for Examining breast cancer screening recommendations in Canada: The projected resource impact of screening among women aged 40–49 [file sj-docx-1-msc-10.1177_09691413241267845.docx]

**Examining breast cancer screening recommendations in Canada: The projected resource impact of screening among women ages 40-49**

Robert B. Basmadjian^1^ PhD, Yibing Ruan^1^ PhD, John M. Hutchinson^1^ BSc, Matthew T. Warkentin^1^ PhD, Oguzhan Alagoz^2,3^ PhD, Andrew Coldman^4^ PhD, Darren R. Brenner^1,5^ PhD

^1^Department of Oncology, Cumming School of Medicine, University of Calgary, Calgary, Alberta, Canada

^2^Department of Industrial and Systems Engineering, University of Wisconsin-Madison, Madison, Wisconsin, USA

^3^Carbone Cancer Center, University of Wisconsin-Madison, Madison, Wisconsin, USA

^4^British Columbia Cancer Control Research, Vancouver, British Columbia, Canada

^5^Department of Community Health Sciences, Cumming School of Medicine, University of Calgary, Calgary, Alberta, Canada

Corresponding Author:

Darren R. Brenner

Department of Oncology and Community Health Sciences

Cumming School of Medicine, University of Calgary

Health Research Innovation Centre Room 2AA21

3230 Hospital Dr NW, Calgary, AB

T2N 4Z6

[darren.brenner@ucalgary.ca](mailto:darren.brenner@ucalgary.ca)

The authors declare no potential conflicts of interest.

**Supplement File**

Table S1. Total number of outcome events among all women in Canada from 2024 to 2043 by screening scenario and target participation rate.

Table S2. Total number of additional outcome events among all women in Canada from 2024 to 2043 in the A40, B40, A45, and B45 scenarios, by target participation rate.

Figure S1. Schematic diagram of OncoSim-Breast model.

Figures S2a-h. Annual number of women screened, number of screens, number of abnormal screening recalls without cancer, number of biopsies, number of negative biopsies, number of invasive breast cancers, number of breast cancers detected by screening, and number of breast cancer deaths between 2015-2050 among all Canadian women the target participation rate of the A40, B40, A45, and B45 scenarios is 50%.

Figures S3a-h. Annual number of women screened, number of screens, number of abnormal screening recalls without cancer, number of biopsies, number of negative biopsies, number of invasive breast cancers, number of breast cancers detected by screening, and number of breast cancer deaths between 2015-2050 among all Canadian women the target participation rate of the A40, B40, A45, and B45 scenarios is 70%.

Figures S4a-h. Annual number of women screened, number of screens, number of abnormal screening recalls without cancer, number of biopsies, number of negative biopsies, number of invasive breast cancers, number of breast cancers detected by screening, and number of breast cancer deaths between 2015-2050 among all Canadian women the target participation rate of the A40, B40, A45, and B45 scenarios is 100%.

Table S1. Total number of outcome events among all women in Canada from 2024 to 2043 by screening scenario and target participation rate.

| **Scenario** | **Frequency** | **Minimum Screening Age** | **Participation Rate (%)** | **Number Of Women Screened** | **Screens** | **False Positives** | **Biopsies** | **Negative Biopsies** | **Invasive Breast Cancers** | **Breast Cancers Detected by Screening** | **Cancer Deaths** |
| --- | --- | --- | --- | --- | --- | --- | --- | --- | --- | --- | --- |
| **Status Quo** | Biennial | 50 | 25 | 5,910,283.27 | 69,573,860.77 | 5,461,198.58 | 939,498.12 | 638,960.23 | 660,943.21 | 300,537.89 | 125,835.48 |
| **A40_50** | Annual | 40 | 50 | 6,684,096.12 | 78,816,485.21 | 6,185,563.58 | 1,036,899.68 | 723,710.94 | 662,747.02 | 313,188.75 | 124,933.58 |
| **B40_50** | Biennial | 40 | 50 | 6,684,096.12 | 74,373,189.55 | 5,874,532.88 | 994,839.23 | 687,320.35 | 661,355.12 | 307,518.88 | 125,253.96 |
| **A45_50** | Annual | 45 | 50 | 6,305,808.91 | 74,180,112.58 | 5,823,188.77 | 989,507.72 | 681,313.09 | 661,968.96 | 308,194.63 | 125,297.04 |
| **B45_50** | Biennial | 45 | 50 | 6,305,808.91 | 71,972,881.71 | 5,668,682.61 | 967,693.66 | 663,235.87 | 661,185.51 | 304,457.80 | 125,399.34 |
| **A40_70** | Annual | 40 | 70 | 7,294,293.22 | 86,140,745.80 | 6,759,281.53 | 1,113,781.38 | 790,835.94 | 664,085.06 | 322,945.44 | 124,109.76 |
| **B40_70** | Biennial | 40 | 70 | 7,294,293.22 | 78,173,442.73 | 6,201,570.31 | 1,038,640.55 | 725,583.73 | 661,734.73 | 313,056.83 | 124,739.74 |
| **A45_70** | Annual | 45 | 70 | 6,617,988.07 | 77,832,308.49 | 6,110,060.40 | 1,028,975.79 | 714,877.07 | 662,790.09 | 314,098.72 | 124,790.89 |
| **B45_70** | Biennial | 45 | 70 | 6,617,988.07 | 73,872,537.16 | 5,832,876.41 | 989,935.80 | 682,446.54 | 661,330.89 | 307,489.26 | 125,111.27 |
| **A40_100** | Annual | 40 | 100 | 8,213,339.18 | 97,111,769.36 | 7,619,157.77 | 1,230,456.90 | 891,441.46 | 666,494.62 | 339,015.44 | 122,976.32 |
| **B40_100** | Biennial | 40 | 100 | 8,213,339.18 | 83,872,048.31 | 6,692,377.30 | 1,105,331.67 | 783,008.14 | 662,674.32 | 322,323.53 | 123,891.68 |
| **A45_100** | Annual | 45 | 100 | 7,087,760.42 | 83,305,952.86 | 6,540,192.74 | 1,088,705.28 | 765,202.55 | 664,206.21 | 323,502.73 | 124,112.45 |
| **B45_100** | Biennial | 45 | 100 | 7,087,760.42 | 76,722,202.06 | 6,079,330.19 | 1,023,711.16 | 711,281.63 | 661,896.27 | 312,429.53 | 124,610.51 |

Table S2. Total number of additional outcome events among all women in Canada from 2024 to 2043 in the A40, B40, A45, and B45 scenarios, by target participation rate (corresponds to Figures 1a-f in main text).

| **Scenario** | **Frequency** | **Minimum Screening Age** | **Participation Rate (%)** | **Number Of Women Screened** | **Screens** | **False Positives** | **Biopsies** | **Negative Biopsies** | **Invasive Breast Cancers** | **Breast Cancers Detected by Screening** | **Cancer Deaths** |
| --- | --- | --- | --- | --- | --- | --- | --- | --- | --- | --- | --- |
| **A40_50** | Annual | 40 | 50 | 773,812.85 | 9,242,624.44 | 724,365.00 | 97,401.56 | 84,750.70 | 1,803.80 | 12,650.85 | -901.90 |
| **B40_50** | Biennial | 40 | 50 | 773,812.85 | 4,799,328.78 | 413,334.30 | 55,341.10 | 48,360.11 | 411.91 | 6,980.99 | -581.52 |
| **A45_50** | Annual | 45 | 50 | 395,525.64 | 4,606,251.81 | 361,990.19 | 50,009.59 | 42,352.85 | 1,025.75 | 7,656.74 | -538.45 |
| **B45_50** | Biennial | 45 | 50 | 395,525.64 | 2,399,020.94 | 207,484.03 | 28,195.54 | 24,275.63 | 242.30 | 3,919.91 | -436.14 |
| **A40_70** | Annual | 40 | 70 | 1,384,009.95 | 16,566,885.03 | 1,298,082.95 | 174,283.25 | 151,875.70 | 3,141.85 | 22,407.55 | -1,725.73 |
| **B40_70** | Biennial | 40 | 70 | 1,384,009.95 | 8,599,581.96 | 740,371.73 | 99,142.43 | 86,623.49 | 791.52 | 12,518.93 | -1,095.74 |
| **A45_70** | Annual | 45 | 70 | 707,704.80 | 8,258,447.72 | 648,861.82 | 89,477.67 | 75,916.83 | 1,846.88 | 13,560.83 | -1,044.59 |
| **B45_70** | Biennial | 45 | 70 | 707,704.80 | 4,298,676.39 | 371,677.83 | 50,437.68 | 43,486.31 | 387.68 | 6,951.37 | -724.21 |
| **A40_100** | Annual | 40 | 100 | 2,303,055.91 | 27,537,908.59 | 2,157,959.19 | 290,958.78 | 252,481.23 | 5,551.41 | 38,477.55 | -2,859.16 |
| **B40_100** | Biennial | 40 | 100 | 2,303,055.91 | 14,298,187.54 | 1,231,178.72 | 165,833.55 | 144,047.91 | 1,731.11 | 21,785.64 | -1,943.80 |
| **A45_100** | Annual | 45 | 100 | 1,177,477.15 | 13,732,092.09 | 1,078,994.16 | 149,207.16 | 126,242.32 | 3,263.00 | 22,964.84 | -1,723.04 |
| **B45_100** | Biennial | 45 | 100 | 1,177,477.15 | 7,148,341.29 | 618,131.61 | 84,213.04 | 72,321.40 | 953.05 | 11,891.64 | -1,224.97 |


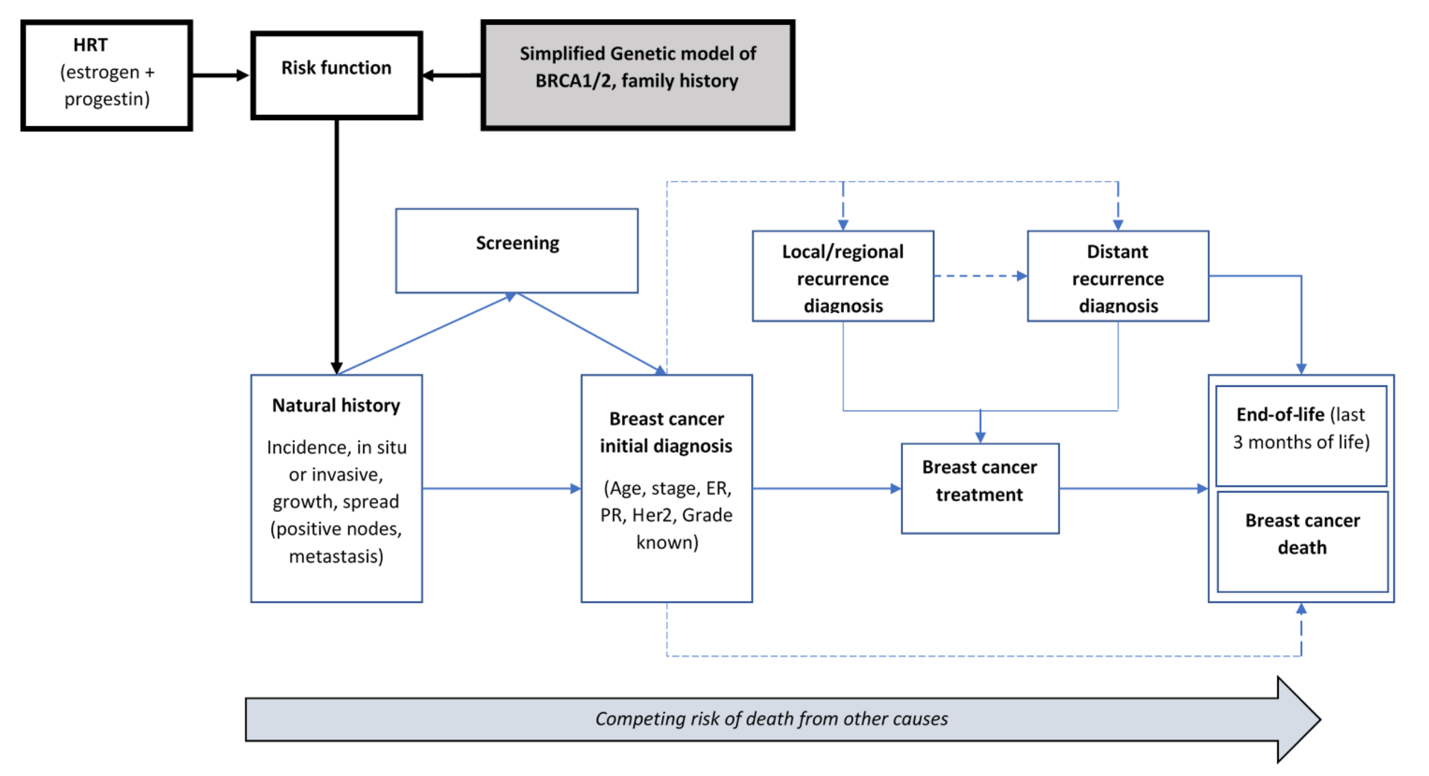


Figure S1. Schematic diagram of OncoSim-Breast model.

Figures S2a-h. Annual number of women screened, number of screens, number of abnormal screening recalls without cancer, number of biopsies, number of negative biopsies, number of invasive breast cancers, number of breast cancers detected by screening, and number of breast cancer deaths between 2015-2050 among all Canadian women the target participation rate of the A40, B40, A45, and B45 scenarios is 50%.

Figures S3a-h. Annual number of women screened, number of screens, number of abnormal screening recalls without cancer, number of biopsies, number of negative biopsies, number of invasive breast cancers, number of breast cancers detected by screening, and number of breast cancer deaths between 2015-2050 among all Canadian women the target participation rate of the A40, B40, A45, and B45 scenarios is 70%.

Figures S4a-h. Annual number of women screened, number of screens, number of abnormal screening recalls without cancer, number of biopsies, number of negative biopsies, number of invasive breast cancers, number of breast cancers detected by screening, and number of breast cancer deaths between 2015-2050 among all Canadian women the target participation rate of the A40, B40, A45, and B45 scenarios is 100%.
